# [3+2] Cycloaddition to a Chiral 5-Methylene-1,3-dioxolan-4-one and Pyrolysis of the Spiro Adducts

**DOI:** 10.3390/molecules30061246

**Published:** 2025-03-10

**Authors:** R. Alan Aitken, Lynn A. Power, Alexandra M. Z. Slawin

**Affiliations:** EaStCHEM School of Chemistry, University of St. Andrews, North Haugh, St. Andrews, Fife KY16 9ST, UK

**Keywords:** methylenedioxolanone, [3+2] cycloaddition, flash vacuum pyrolysis, spiro heterocycles, NMR data, X-ray structure

## Abstract

The [3+2] cycloaddition chemistry of (2*S*)-5-methylene-2-*t*-butyl-1,3-dioxolan-4-one, derived from lactic acid, has been examined, and spiro adducts have been obtained with benzonitrile oxide, acetonitrile oxide, diazomethane and diphenyldiazomethane. The structure and absolute stereochemistry of the benzonitrile oxide adduct has been confirmed by X-ray diffraction, and all the adducts have been fully characterised by ^1^H and ^13^C NMR. Attempted cycloaddition with a nitrile sulfide, a nitrile imine and azides failed. Pyrolysis results in a range of novel gas-phase reactions, with the nitrile oxide adducts giving pivalaldehyde, CO_2_, the nitrile and ketene, the diazomethane adduct losing only N_2_ to give a cyclopropane-fused dioxolanone, and the diphenylcyclopropane derived from diphenyldiazomethane giving mainly benzophenone in a sequence involving the loss of pivalaldehyde and methyleneketene.

## 1. Introduction

**Scheme 1:**
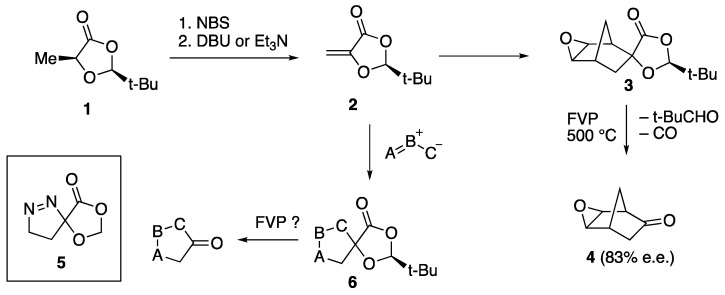
Opportunities for cycloaddition chemistry to the double bond of **2**, and the only previous methylenedioxolanone [3+2] cycloadduct.

Spiro heterocycles have been of considerable recent interest due to their rigidly defined geometry and potential as scaffolds in medicinal chemistry [1,2,3]. In previous work, it has been shown that chiral dioxolanones such as compound **1** (Scheme 1) readily derived from (*S*)-lactic acid have considerable value in a range of asymmetric synthesis methods [4,5]. Some time ago, we showed that deprotection by flash vacuum pyrolysis (FVP) resulted in the loss of pivalaldehyde and CO from substituted dioxolanones to give ketones [6], thus making the starting dioxolanone **1** a chiral acetyl anion equivalent [7]. Bromination of **1** followed by dehydrobromination affords the 5-methylene compound **2** [8], and this has been found to undergo a range of Diels–Alder reactions [9,10,11,12]. We recently described the Diels–Alder reaction of **2** with several 1,3-dienes, and again, deprotection of the adducts using FVP was found to afford ketones in some cases. This is illustrated by the reaction of epoxide **3**, formed by the cycloaddition of **2** with cyclopentadiene followed by epoxidation, to give the epoxynorbornanone **4** in high e.e. [13]. Overall, compound **2** acts as a chiral ketene equivalent.

As far as we are aware, the [3+2] cycloaddition chemistry of chiral dioxolanone **2** has never been investigated, and indeed we are only aware of a single example of [3+2] cycloaddition to any 5-alkylidene-1,3-dioxolan-4-one, compound **5** formed from the 2-unsubstituted dioxolanone and diazomethane [14]. The historical development of the [3+2] cycloaddition reaction and its mechanistic understanding has been chronicled in a recent review [15]. Methylenedioxolanone **2** is a particularly interesting 2π-component for [3+2] cycloaddition, since the bulky *tert*-butyl group introduces a strong facial bias and the carbonyl and enol-ether functions exert opposing polarising effects on the C=C double bond. Based on previous studies of its [4+2] cycloaddition with both homo- and hetero-dienes [10], it is expected that addition will occur on the face remote from the *tert*-butyl and that the polarising effect of the enol ether will dominate, causing the “negative” end of any TAC to add at the ring carbon. In this paper, we describe the cycloaddition of **2** with a range of three-atom components (TACs, formerly known as 1,3-dipoles [16]) to give spiro heterocycles of general structure **6,** and FVP of these adducts.

## 2. Results and Discussion

### 2.1. [3+2] Cycloaddition to Methylenedioxolanone **2**

**Scheme 2:**
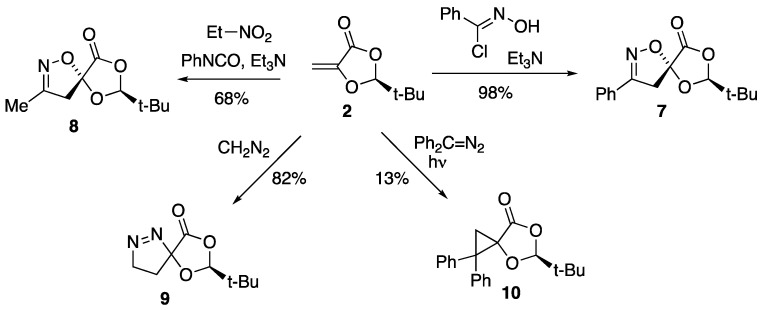
Formation of [3+2] cycloadducts from **2**.

Treatment of compound **2** and benzhydroximoyl chloride in diethyl ether with triethylamine [17] at 0 °C for 30 min followed by room temperature for 30 min gave the crystalline benzonitrile oxide adduct **7** in almost quantitative yield as a single stereoisomer (Scheme 2). This showed the expected NMR signals for a diastereotopic CH_2_ group [δ_H_ 3.52, 3.94 (^2^*J* 17.7 Hz); δ_C_ 42.3], as well as a signal for the spiro carbon [δ_C_ 105.2]. The corresponding acetonitrile oxide adduct **8** was also readily prepared by addition of a solution of **2** and phenyl isocyanate in toluene to a cooled solution of nitroethane in toluene containing a catalytic quantity of triethylamine [18]. The product was isolated as an oil in moderate yield, again showing characteristic NMR signals for the diastereotopic CH_2_ group [δ_H_ 3.07, 3.55 (^2^*J* 18.2 Hz), δ_C_ 91.0], this time with additional four-bond coupling to the methyl group (^4^*J* 0.8 Hz) and a signal for the spiro carbon [δ_C_ 91.0]. Again, the compound was obtained as a single stereoisomer, and from the similarity of the NMR data it seemed clear that **7** and **8** were formed with the same regio- and stereo-selectivity.

**Figure 1:**
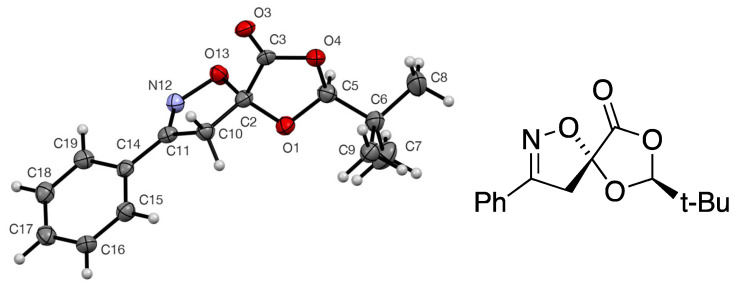
X-ray structure of **7** with thermal ellipsoids (50%) and crystallographic numbering system used.

The crystals of compound **7** were suitable for X-ray structure determination, and this gave the molecular structure shown (Figure 1, Table 1), in which the TAC has added to the face of the C=C double bond away from the bulky *tert*-butyl group and with the more electronegative end of the TAC adding to the ring carbon of the C=C double bond. It is thus clear that this 2-π component is exhibiting the same facial and regio-selectivity in [3+2] cycloaddition as expected from the earlier studies on its [4+2] cycloaddition with heterodienes [10]. There were no significant intermolecular interactions in the crystal structure. We assume that the acetonitrile oxide adduct **8** has the corresponding stereochemistry, as depicted in Scheme 2.

Reaction with related TACs was now examined, but the dioxolanone **2** failed to react with either benzonitrile sulfide (formed by thermolysis of 5-phenyl-1,3,4-oxathiazol-2-one [19]) or with diphenylnitrile imine (formed by triethylamine treatment of α-chlorobenzaldehyde phenylhydrazone [20]).

As mentioned earlier, the one literature example of [3+2] cycloaddition to a methylenedioxolanone is compound **5**, formed from diazomethane [14]. Compound **2** was therefore treated with an ethereal solution of diazomethane, and the expected adduct **9** was formed in good yield. This showed distinctive NMR signals for two adjacent but strongly differentiated CH_2_ groups, corresponding to C–CH_2_ [δ_H_ 1.73, 2.20; δ_C_ 24.6] and =N–CH_2_ [δ_H_ 4.62, 4.87; δ_C_ 77.3], together with a signal for the spiro carbon [δ_C_ 114.4]. Compound **2** failed to react with diphenyldiazomethane under thermal conditions, but upon photolysis a new product was formed in low yield which turned out to be the cyclopropane **10**. This showed an unexpectedly complex NMR pattern, with restricted rotation in the sterically hindered phenyl groups leading to separate signals for some of the normally equivalent aromatic carbons, [δ_C_ 6 × CH, 2 × 2CH].

The reactivity of dioxolanone **2** with ethyl diazoacetate, ethyl azidoformate and phenyl azide was also examined under both thermal and photochemical conditions, but the starting material was recovered unchanged in all cases. Perhaps owing to steric hindrance and the push–pull substituents, this seems to be a rather reluctant 2-π electron component.

### 2.2. Flash Vacuum Pyrolysis of the Spiro Cycloadducts

Having obtained the four spiro cycloadducts **7**–**10** with novel structures, we were interested to examine their thermal decomposition. Based on our previous work [13], this was initially expected to yield cyclic ketones **6** by loss of pivalaldehyde and carbon monoxide (Scheme 1).

**Scheme 3:**
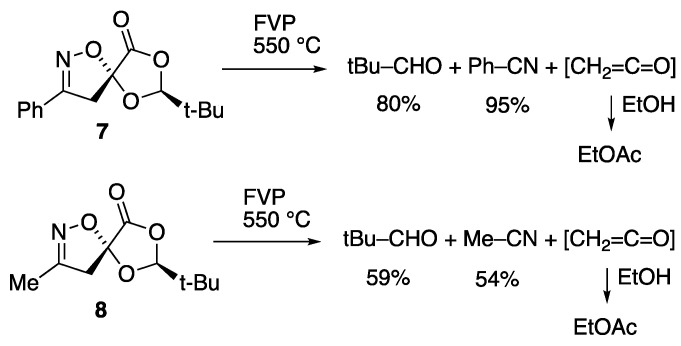
Pyrolysis products from the nitrile oxide adducts.

Subjecting the benzonitrile oxide adduct **7** to FVP led to complete reaction at a temperature of 550 °C (Scheme 3), to give high yields of benzonitrile and pivalaldehyde recovered from the cold trap after warming to room temperature, identified by ^1^H and ^13^C NMR spectroscopy (see Supplementary Materials).

Mechanistic considerations led to the suspicion that ketene might also be produced, and this was confirmed by adding a small amount of ethanol to the trap while still cold, which led to the formation of ethyl acetate upon warming up. FVP of the acetonitrile oxide adduct **8** also led to complete reaction at the same temperature, giving acetonitrile, pivalaldehyde and ketene.

**Scheme 4:**
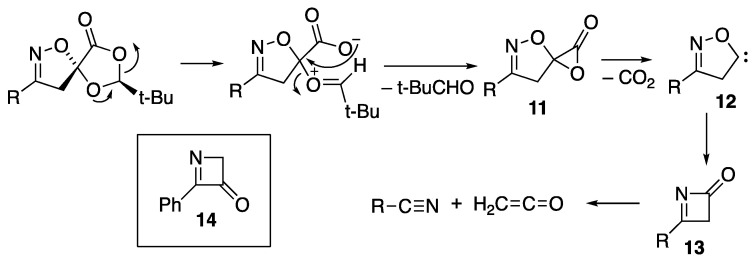
Proposed mechanism for the pyrolysis of **7** and **8**.

Based on our previous work [13], we believe that this process is initiated by ring opening to an oxonium carboxylate which then loses pivalaldehyde to form the spiro α-lactone **11** (Scheme 4). Rather than undergoing loss of CO to form an isoxazolinone, as observed, for example, in the conversion of **3** into **4**, it is apparently more favourable for this adduct to lose CO_2_ to give the heterocyclic carbene **12**, which readily undergoes oxa-carbene rearrangement, resulting in ring contraction to the 3*H*-azetin-2-one **13**, which then fragments to the nitrile and ketene.

Although such azetinones are unknown, it is interesting to note that there is one claim to have generated the isomeric 4-phenyl-2*H*-azetin-3-one **14** during the photolysis of benzonitrile in the presence of 1,3-dioxole followed by subsequent hydrolysis and dehydration [21]. However, the data presented, including in particular a ^1^H NMR signal at δ_H_ 7.9 ppm attributed to CH_2_ with no corresponding signal apparent in the ^13^C NMR spectrum, do not support the structure shown.

**Scheme 5:**
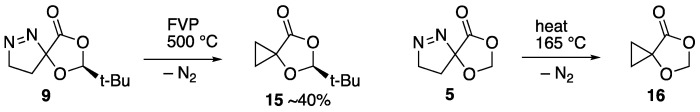
FVP behaviour of spiro pyrazoline–dioxolanone **9** and comparison with **5** [14].

We now turned to the diazomethane adduct **9**, and it was found to undergo complete reaction upon FVP at 500 °C. However, rather than any process involving the dioxolanone, it was clear that there had simply been extrusion of N_2_ to give the spiro cyclopropane–dioxolanone **15** (Scheme 5). This was obtained in low yield as an oil and gave satisfactory HRMS data, as well as distinctive low-frequency signals for the two non-equivalent CH_2_ groups in its NMR spectra [δ_H_ 1.14–1.32; δ_C_ 11.3, 13.2] and a signal for the spiro carbon [δ_C_ 60.0]. This behaviour is in good agreement with that observed for the 2-unsubstituted analogue **5**, which was reported to lose N_2_ to afford **16** upon being slowly added to a flask containing pieces of porcelain heated at 165 °C [14].

**Scheme 6:**
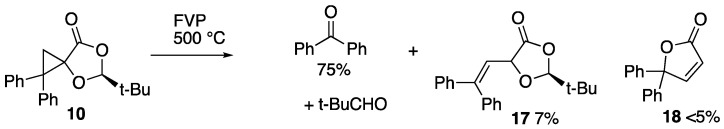
FVP of the spiro cyclopropane–dioxolanone **10**.

Finally, the thermal reactivity of spiro diphenylcyclopropane–dioxolanone **10** was examined. Reaction was again complete upon FVP at a furnace temperature of 500 °C, and the NMR analysis of the product showed the presence of pivalaldehyde and several phenyl-containing compounds. The latter were separated by preparative TLC to give a main fraction which crystallised and was found to consist mainly of benzophenone (75%), together with a little starting material and a new compound in low yield which we believe to be the diphenylvinyldioxolanone **17**, isomeric with **10** (Scheme 6). This exhibited distinctive mutually coupled doublets in the ^1^H NMR spectrum [δ_H_ 5.12, 6.12 (^3^*J* 10 Hz)] as well as the expected 1H and 9H singlets but, due to the low yield, further characterisation was not possible. A second, less polar fraction gave distinctive signals for 4,4-diphenylbutenolide **18** [22], but this was only formed in less than 5% yield.

**Scheme 7:**
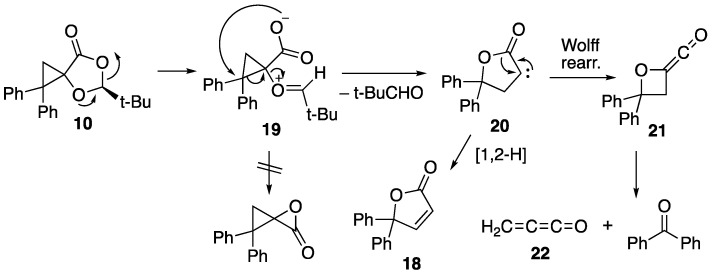
Proposed mechanism for the formation of benzophenone from FVP of **10**.

While the minor product **17** is a result of simple thermal ring-opening of the cyclopropane, we propose that both the major product benzophenone and **18** are formed by the mechanism shown in Scheme 7.

**Scheme 8:**
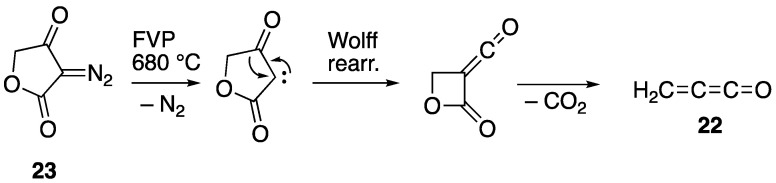
Similar FVP reaction previously used to generate **22** [22].

In this scheme, the normal fragmentation initially gives the oxonium carboxylate **19** but, rather than cyclising with loss of pivalaldehyde to give the extremely strained α-lactone, as happens for the nitrile oxide adducts **7** and **8**, this rearranges by attack of the carboxylate on the electron-poor diphenylcyclopropane carbon, resulting in loss of pivalaldehyde and formation of the butyrolactone carbene **20**. As a minor process, this can undergo a 1,2-hydrogen shift to form the stable butenolide **18**. However, it appears that it prefers to undergo Wolff rearrangement to give the oxetanylideneketene **21,** which is set up to undergo cycloreversion to form benzophenone and the unstable methyleneketene (propadienone) **22**. Compound **22**, when generated using FVP, cannot be isolated under normal conditions and forms a glassy polymer [23], which we assume is its fate here. The formation of **22** in the present reaction is rather similar to the method used by Chapman to generate it for low-temperature matrix IR studies, starting from the diazo compound **23** (Scheme 8) [24].

### 2.3. NMR Data for the Spiro Compounds

**Figure 2:**
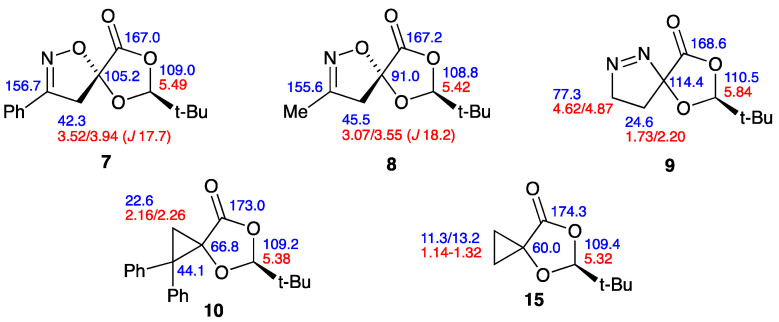
Compilation of NMR data for new spiro dioxolanones (blue: δ_C_; red: δ_H_).

**Figure 3:**
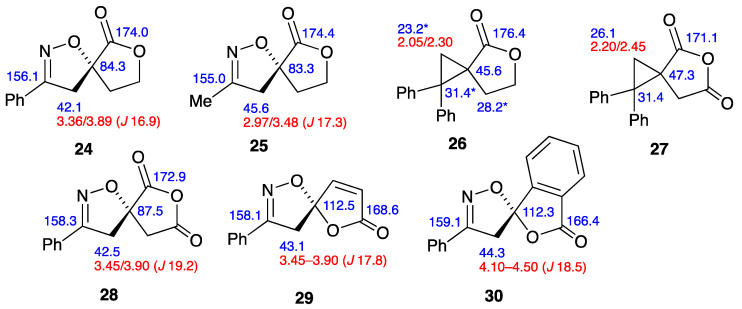
NMR data for similar spiro heterocyclic compounds (blue: δ_C_; red: δ_H_; * indicates assignments may be interchanged).

In the course of this work, we have obtained products containing a range of novel spiro heterocyclic ring systems. For these, the ^1^H and ^13^C NMR data have been unambiguously assigned including, where necessary, using HSQC and HMBC methods. These data are combined and presented in pictorial form in Figure 2. While these data form a self-consistent pattern, it was realised that there are few closely similar spiro systems in the literature. We have therefore compiled the published data for the most closely similar systems, and this is presented in Figure 3.

As mentioned earlier, the only spiro dioxolanone product previously formed by [3+2] cycloaddition is compound **5**, but due to the early date of its preparation there are no NMR data either for it nor its cyclopropane pyrolysis product **16**. The most closely analogous structures for which there are NMR data are formed from α-methylenebutyrolactone. Thus, its benzonitrile oxide adduct **24** was reported in 1995 [25], while the acetonitrile oxide adduct **25** was reported in 2015 [26]. It can be seen that there is good agreement with the data for **7** and **8** in both cases.

Similarly, the diphenylcyclopropane compound **26** obtained from α-methylenebutyrolactone [27] provides a good comparison with the data for **10**, although there is some ambiguity in the assignment of the marked signals, which were not assigned in the original publication. Further relevant spiro compounds are the diphenylcyclopropane **27** [28] and benzonitrile oxide adduct **28** [29], both derived from itaconic anhydride. Further, more diverse structures for comparison with the data of the benzonitrile oxide adduct **7** are **29**, derived from “protoanemonin” [30], and **30**, derived from 3-methylenephthalide [31]. Overall, we hope that this compilation of data may provide a useful reference for future workers in the field.

## 3. Experimental Procedure

### 3.1. General Experimental Details

NMR spectra were recorded for solutions in CDCl_3_ using Bruker instruments, and chemical shifts are given in ppm to high frequency from Me_4_Si with coupling constants *J* in Hz. On ^1^H NMR spectra, signals at 0.0 and 7.26 are due to internal Me_4_Si and residual CHCl_3_, respectively. The ^13^C NMR spectra are referenced to the solvent signal at 77.0. IR spectra were recorded on a Perkin Elmer 1420 instrument. Elemental analysis was conducted using a Carlo Erba CHNS analyser. Mass spectra were obtained using a Micromass instrument, and the ionisation method used is noted in each case. Column chromatography was carried out using silica gel of 40–63 μm particle size, and preparative TLC was carried out using 1.0 mm layers of Merck alumina 60G containing 0.5% Woelm fluorescent green indicator on glass plates. Melting points were recorded on a Gallenkamp 50W melting point apparatus or a Reichert hot-stage microscope. Optical rotation measurements were made using an Optical Activity 1000 polarimeter and are given in units of 10^−1^ deg cm^2^ g^−1^.

Flash vacuum pyrolysis (FVP) was carried out in a conventional flow system by subliming the starting material through a horizontal quartz tube (30 × 2.5 cm) externally heated by a tube furnace to 500–550 °C and maintained at a pressure of 2–5 × 10^−2^ Torr by a rotary vacuum pump. Products were collected in a liquid-N_2_-cooled U-shaped trap and purified as noted.

General organic and inorganic reagents and solvents were obtained from standard suppliers and used as received. Dry diethyl ether was prepared by storage over sodium wire. The starting materials (2*R*)-2-t-butyl-5-methylene-1,3-dioxolan-4-one **2** [8], benzhydroximoyl chloride [32], diazomethane [33] and diphenyldiazomethane [34] were prepared by the reported methods.

### 3.2. Preparation of [3+2] Cycloadducts of (2R)-2-t-Butyl-5-methylene-1,3-dioxolan-4-one ***2***

#### 3.2.1. Preparation of (2*S*,5*R*)-Spiro[2-*t*-butyl-1,3-dioxolan-4-one-5,5′-4′,5′-dihydro-3′-phenylisoxazole] **7**

A solution of **2** (1.50 g, 9 mmol) and benzhydroximoyl chloride (1.50 g, 9.6 mmol) was stirred in Et_2_O (50 cm^3^) at 0 °C, while a solution of triethylamine (1.01 g, 10 mmol) in Et_2_O (8 cm^3^) was added dropwise over 30 min. The mixture was stirred for a further 30 min at room temperature. The precipitate, triethylamine hydrochloride, was filtered off and the filtrate was washed with water. The ethereal layer was dried and evaporated to give a pale yellow solid. Recrystallisation using Et_2_O/CH_2_Cl_2_/hexane gave the pure product **7** (2.43 g, 98%) as colourless needles, mp 107–109 °C; [α]_D_ +1.0 (c = 1.0, CH_2_Cl_2_); (Found: C, 65.5; H, 6.3; N, 5.2. C_15_H_17_NO_4_ requires C, 65.4; H, 6.2; N, 5.1%); ν_max_/cm^−1^ 1804 (C=O), 1349, 1256, 1198, 1187, 1177, 1065, 1011, 962 and 853; δ_H_ 1.00 (9H, s, t-Bu), 3.52 and 3.94 (2H, AB pattern, *J* 17.7, 4′-CH_2_), 5.49 (1H, s, CH-t-Bu), 7.41–7.49 (3H, m, Ph) and 7.66–7.72 (2H, m, Ph); δ_C_ 23.1 (3CH_3_, t-Bu), 34.3 (C, CMe_3_), 42.3 (4′-CH_2_), 105.2 (5/5′-C), 109.0 (2-CH), 127.0 (2CH, Ph), 127.8 (C, Ph C-1), 129.0 (2 CH, Ph), 131.1 (CH, Ph C-4), 156.7 (C=N) and 167.0 (C=O); *m*/*z* (ES^+^) 298.21, (M+Na^+^, 100%).

#### 3.2.2. Preparation of (2*S*,5*R*)-Spiro[2-*t*-butyl-1,3-dioxolan-4-one-5,5′-4′,5′-dihydro-3′-methylisoxazole] **8**

A solution of nitroethane (0.83 g, 11 mmol) and triethylamine (1 drop) in toluene (1 cm^3^) was stirred at room temperature, and a solution of phenyl isocyanate (1.3 g, 11 mmol) and **82** (1.6 g, 10 mmol) in toluene (1.2 cm^3^) was added dropwise over 1 h. An initial colour change from colourless to canary yellow along with rapid heating was observed, and a cold water bath was used to prevent boiling of the solvent. Once the reaction was deemed complete, the precipitate was filtered off and the solvent removed under vacuum. The resulting oil was taken up in hexane and CH_2_Cl_2_ and left to crystallise. The solid impurity was filtered off and the filtrate evaporated to give product **8** as an orange oil (1.46 g, 68%). [α]_D_ +20.65 (c = 1.22, CH_2_Cl_2_). HRMS *m*/*z* (CI): found 214.1085. C_10_H_16_NO_4_ (M+H) requires 214.1079; ν_max_/cm^−1^ 1810 (C=O), 1763, 1720, 1299, 1233, 1164, 1072, 983 and 848; δ_H_ 0.97 (9H, s, t-Bu), 2.11 (3H, dd, *J* 1.1, 0.8, 3′-CH_3_), 3.07 (1H, half AB pattern of q, *J* 18.2, 0.8, 4′-CH_2_), 3.55 (1H, half AB pattern of q, *J* 18.2, 1.1, 4′-CH_2_) and 5.42 (1H, s, CH-t-Bu); δ_C_ 12.7 (3′-CH_3_), 23.1 (3CH_3_, t-Bu), 34.2 (C, CMe_3_), 45.5 (4′-CH_2_), 91.0 (5/5′-C), 108.8 (2-CH), 155.6 (C=N) and 167.2 (C=O); *m*/*z* (CI^+^) 214.11 (M+H^+^, 8%) and 128.03 (M^+^–Bu^t^CHO, 100%).

#### 3.2.3. Preparation of (2*S*)-Spiro[2-*t*-butyl-1,3-dioxolan-4-one-5,3′-3′,4′-dihydro-5*H*-pyrazole] **9**

The dioxolanone **2** (0.6 g, 3.8 mmol) was placed in a conical flask [**important**: no ground glass joints] with Et_2_O (10 cm^3^) in an ice bath, and an excess of diazomethane/Et_2_O [32] was distilled into the solution. Once this was complete, the reaction mixture was allowed to warm up to room temperature and stirred overnight to allow any unreacted gas to disperse. The solvent was then removed to yield the desired product **9** as a yellow viscous oil (0.62 g, 82%). HRMS *m*/*z* (CI): found 199.1092. C_9_H_15_N_2_O_3_ (M+H) requires 199.1083; ν_max_/cm^−1^ 1797 (C=O), 1721, 1366, 1289, 1223 and 1158; δ_H_ 1.03 (9H, s, t-Bu), 1.73 (1H, ddd, *J* 13, 9, 6, 4′-CH_2_), 2.20 (1H, ddd, *J* 13, 9, 4, 4′-CH_2_), 4.62 (1H, ddd, *J* 18, 9, 6, 3′-CH_2_), 4.87 (1H, ddd, *J* 18, 9, 4, 3′-CH_2_) and 5.84 (1H, s, CH-t-Bu); δ_C_ 23.1 (3CH_3_, t-Bu), 24.6 (4′-CH_2_), 34.7 (C, CMe_3_), 77.3 (3′-CH_2_), 110.5 (2-CH), 114.4 (5/5′-C) and 168.6 (C=O); and *m*/*z* (CI^+^) 199.11 (M+H^+^, 1%) and 95.02 (M^+^–CO, Bu^t^CHO, 100%).

#### 3.2.4. Preparation of (2*S*)-Spiro[2-*t*-butyl-1,3-dioxolan-4-one-5,1′-2′,2′-diphenylcyclopropane] **10**

The reactants **2** (0.9 g, 5.7 mmol) and diphenyldiazomethane (1.10 g, 5.7 mmol) were stirred in Et_2_O (10 cm^3^) for 5 days and monitored by TLC. No reaction had occurred, so the contents of the reaction vessel were transferred into a quartz tube and irradiated with a 400 W medium-pressure mercury lamp for 2 h. After this time, TLC analysis indicated that a reaction had taken place. The material was separated by flash column chromatography (SiO_2_, Et_2_O/hexane, 12:1) to furnish the product as a yellow/orange oil (0.25 g, 13%); HRMS *m*/*z* (CI): found 323.1642. C_21_H_23_O_3_ (M+H) requires 323.1647 ν_max_/cm^−1^ 3057, 1443, 1320 and 691; δ_H_ 0.96 (9H, s, t-Bu), 2.16 and 2.26 (2H, AB pattern, *J* 6.7, 3′-CH_2_), 5.38 (1H, s, CH-t-Bu), 7.27–7.35 (2H, m, Ph), 7.45–7.52 (3H, m, Ph), 7.56–7.63 (2H, m, Ph) and 7.78–7.84 (3H, m, Ph); δ_C_ 22.6 (3′-CH_2_), 23.2 (3CH_3_, t-Bu), 34.5 (C, CMe_3_), 44.1 (2′-C), 66.8 (5/1′-C), 109.2 (2-CH), 127.0 (CH, Ph), 127.4 (CH, Ph), 128.30 (2CH, Ph), 128.34 (CH, Ph), 128.7 (CH, Ph), 129.0 (CH, Ph), 129.2 (CH, Ph), 130.0 (2CH, Ph), 137.6 (C, Ph C-1), 138.8 (C, Ph C-1) and 173.0 (C=O); *m*/*z* (ES) 345.11 (M+Na^+^, 100%).

### 3.3. Pyrolysis of [3+2] Cycloadducts

#### 3.3.1. Pyrolysis of (2*S*,5*R*)-Spiro[2-*t*-butyl-1,3-dioxolan-4-one-5,5′-4′,5′-dihydro-3′-phenylisoxazole] **7**

FVP of the reactant **7** (0.1109 g, 0.40 mmol) was performed at 550 °C and 4.4 × 10^−1^ Torr. NMR spectroscopic analysis of the cold trap material indicated that no starting material remained. The substances present were pivalaldehyde (80%) [δ_H_ 1.08 (9H, s, t-Bu) and 9.48 (1H, s, CH=O); δ_C_ 23.3, 42.5 and 206.0] and benzonitrile (95%) [δ_H_ 7.46–7.50 (2H, m, Ph) and 7.60–7.69 (3H, m, Ph); δ_C_ 112.4 (C), 118.8 (C≡N), 129.0 (2CH), 132.1 (2CH) and 132.7 (CH)]

The formation of ketene was determined by repeating the pyrolysis and adding a small amount of ethanol directly into the cold trap immediately after isolation of the trap from the vacuum system. This gave ethyl acetate (40%) [δ_H_ 1.26 (3H, t, *J* 7.1, CH_3_), 2.04 (3H, s, CH_3_) and 4.12 (2H, q, *J* 7.1, CH_2_)].

#### 3.3.2. Pyrolysis of (2*S*,5*R*)-Spiro[2-*t*-butyl-1,3-dioxolan-4-one-5,5′-4′,5′-dihydro-3′-methylisoxazole] **8**

FVP of the reactant **8** (0.1454 g, 0.68 mmol) was performed at 550 °C and 4.4 × 10^−1^ Torr. ^1^H NMR spectroscopic analysis of the cold trap material indicated that no starting material remained. The substances present were pivalaldehyde (59%) [δ_H_ 1.08 (9H, s, t-Bu) and 9.48 (1H, s, CH=O)] and acetonitrile (54%) [δ_H_ 2.01 (3H, s, CH_3_)].

As described above, the formation of ketene was demonstrated by addition of a few drops of ethanol to the cold trap following pyrolysis. This gave ethyl acetate (23%) [δ_H_ 1.26 (3H, t, *J* 7.1, CH_3_) 2.04 (3H, s, CH_3_) and 4.12 (2H, q, *J* 7.1, CH_2_)].

#### 3.3.3. Pyrolysis of (2*S*)-Spiro [2-*t*-butyl-1,3-dioxolan-4-one-5,3′-3′,4′-dihydro-5*H*-pyrazole] **9**

FVP of the reactant **9** (0.0367 g, 0.185 mmol) was performed at 500 °C and 7.5 × 10^−1^ Torr. The product obtained was the spiro cyclopropane–dioxolanone **15** (ca. 40%) as an oil; ν_max_/cm^−1^ 1799 (C=O), 1286, 1151 and 1075; HRMS *m*/*z* (CI): found 171.1019. C_9_H_15_O_3_ (M+H) requires 171.1021; δ_H_ 0.99 (9H, s, t-Bu), 1.14–1.32 (4H, m, CH_2_-CH_2_) and 5.32 (1H, s, CH-Bu^t^); δ_C_ 11.3 (CH_2_), 13.2 (CH_2_), 23.3 (3CH_3_, t-Bu), 34.9 (C, CMe_3_), 60.0 (C-5) 109.4 (2-CH) and 174.3 (C=O); *m*/*z* (CI) 171 (M+H^+^, 100%). In addition, there were traces of **9** and pivalaldehyde [δ_H_ 1.08 (9H, s, t-Bu) and 9.48 (1H, s, CH=O)].

#### 3.3.4. Pyrolysis of (2*S*)-Spiro[2-*t*-butyl-1,3-dioxolan-4-one-5,1′-2′,2′-diphenylcyclopropane] **10**

FVP of the reactant **10** (0.08 g, 0.22 mmol) was performed at 500 °C and 4.3 × 10^−1^ Torr. NMR spectroscopic analysis of the cold trap material showed the presence of pivalaldehyde [δ_H_ 1.08 (9H, s, t-Bu) and 9.48 (1H, s, CH=O)] and several phenyl-containing materials. The separation of the non-volatile products by preparative TLC (Et_2_O/ hexane, 1:3) gave as the first fraction 4,4-diphenylbutenolide **18** (<5%) [δ_H_ 6.19 (1H, d, *J* 5.5, 2-CH), 7.94 (1H, d, *J* 5.5, 3-CH)]. The second, much larger fraction was a yellow oil (0.0365 g) which crystallised with time. This consisted, by NMR, mainly of benzophenone (75%) [δ_H_ 7.45–7.50 (4H, m, Ph), 7.56–7.62 (2H, m, Ph C-4) and 7.78–7.82 (4H, m, Ph); δ_C_ 128.3 (4CH, Ph), 130.0 (4CH, Ph), 1324 (2CH, Ph C-4), 137.6 (2C, Ph C-1) and 196.8 (C=O)], a little starting material, and a new product tentatively identified as 2-*tert*-butyl-5-(2,2-diphenylvinyl)-1,3-dioxolan-4-one **17** (7%) [δ_H_ 0.92 (9H, s, t-Bu), 5.12 (1H, d, *J* 9.9, 5-CH), 5.46 (1H, s, 2-CH), 6.12 (1H, d, *J* 9.9, 1′-=CH) and 7.18–7.35 (10H, m, Ph)].

### 3.4. X-Ray Structure Determination of Adduct ***7***

Data were collected on a Bruker SMART diffractometer using graphite-monochromated Mo Kα radiation, *λ* = 0.71073 Å. The data were deposited at the Cambridge Crystallographic Data Centre and can be obtained free of charge via http://www.ccdc.cam.ac.uk/structures. The structure was solved by direct methods and refined by full-matrix least squares against F^2^ (SHELXL, Version 2018/3 [35]).

#### (2*S*,5*R*)-Spiro[2-*t*-butyl-1,3-dioxolan-4-one-5,5′-4′,5′-dihydro-3′-phenylisoxazole] **7**

Crystal data for C_15_H_17_NO_4_, *M* = 275.30, colourless prism, crystal dimensions 0.30 × 0.10 × 0.03 mm, orthorhombic, space group P2_1_2_1_2_1_ (No. 19), *a* = 5.7153(14), *b* = 11.497(3), *c* = 20.903(5) Å, *V* = 1373.5(6) Å^3^, *Z* = 4, *D*_c_ = 1.331 g cm^−3^, *T* = 93(2) K, *R*1 = 0.0513, *Rw*2 = 0.1029 for 2284 reflections with *I* > 2σ(*I*) and 182 variables, CCDC 2409957.

## 4. Conclusions

The first examples of [3+2] cycloaddition to the double bond of (2*S*)-5-methylene-2-*t*-butyl-1,3-dioxolan-4-one have been observed. The compound reacts well with nitrile oxides and diazo compounds but not with a range of other TAC types. NMR data have been documented for the novel spiro ring systems obtained, and the structure has been confirmed in one case by X-ray diffraction. Upon FVP, the adducts undergo a variety of processes involving interesting reactive intermediates whose reactivity has not been examined before. The addition of further types of TAC seems likely to provide an entry to more new chemistry of fused-ring heterocycles, and these results will be reported shortly.

## Figures and Tables

**Table 1:** Heterocyclic ring dimensions for **7** (Å, °).

Bond Lengths				Angles			
C(2)–C(10)	1.480(3)	C(2)–C(3)	1.502(3)	O(13)–C(2)–C(10)	104.98(16)	C(3)–C(2)–O(1)	103.57(16)
C(10)–C(11)	1.487(3)	C(3)–O(3)	1.187(2)	C(2)–C(10)–C(11)	101.08(17)	C(2)–O(1)–C(5)	107.75(16)
C(11)–N(12)	1.264(3)	C(3)–O(4)	1.327(3)	C(10)–C(11)–N(12)	113.4(2)	O(1)–C(5)–O(4)	104.86(17)
N(12)–O(13)	1.421(2)	O(4)–C(5)	1.435(3)	C(11)–N(12)–O(13)	108.29(16)	C(5)–O(4)–C(3)	108.44(17)
O(13)–C(2)	1.420(3)	C(5)–O(1)	1.405(3)	N(12)–O(13)–C(2)	108.62(15)	O(4)–C(3)–C(2)	108.02(19)
		O(1)–C(2)	1.383(3)	O(13)–C(2)–C(3)	106.59(17)	O(1)–C(2)–C(10)	112.03(18)

## Data Availability

The research data supporting this publication (original NMR files) can be accessed at https://doi.org/10.17630/49397269-7e5e-4183-89cc-1dab0c7b16b8.

## Supplementary Materials

The following supporting information can be downloaded at: www.mdpi.com/article/10.3390/molecules30061246/s1, Figures S1–S17: NMR spectra of new compounds.
