# Supplementary material for: [3+2] Cycloaddition to a Chiral 5-Methylene-1,3-dioxolan-4-one and Pyrolysis of the Spiro Adducts
Source: Molecules. 2025 Mar 10;30(6):1246. doi: 10.3390/molecules30061246 (PMC11946748; doi:10.3390/molecules30061246)

[3+2] Cycloaddition to a Chiral 5-Methylene-1,3-dioxolan-4-one and Pyrolysis of the Spiro Adducts

R. Alan Aitken*, Lynn A. Power and Alexandra M. Z. Slawin
EaStCHEM School of Chemistry, University of St Andrews, North Haugh, St Andrews, Fife, KY16 9ST, UK.

Supplementary Material	Figure
^{1}H NMR and DEPTQ ^{13}C NMR spectra of **7**	S1, S2
^{1}H NMR and DEPTQ ^{13}C NMR spectra of **8**	S3, S4
^{1}H NMR and DEPTQ ^{13}C NMR spectra of **9**	S5, S6
^{1}H NMR and DEPTQ ^{13}C NMR spectra of **10**	S7, S8
^{1}H NMR and DEPTQ ^{13}C NMR spectra of pyrolysate from **7**	S9, S10, S11
^{1}H NMR spectrum of pyrolysate from **8**	S12
^{1}H NMR and DEPTQ ^{13}C NMR spectra of pyrolysate from **9** containing **15**	S13, S14
^{1}H NMR and DEPTQ ^{13}C NMR spectra of pyrolysate from **10**	S15, S16, S17

Figure S1. 300 MHz ^{1}H NMR spectrum of **7**

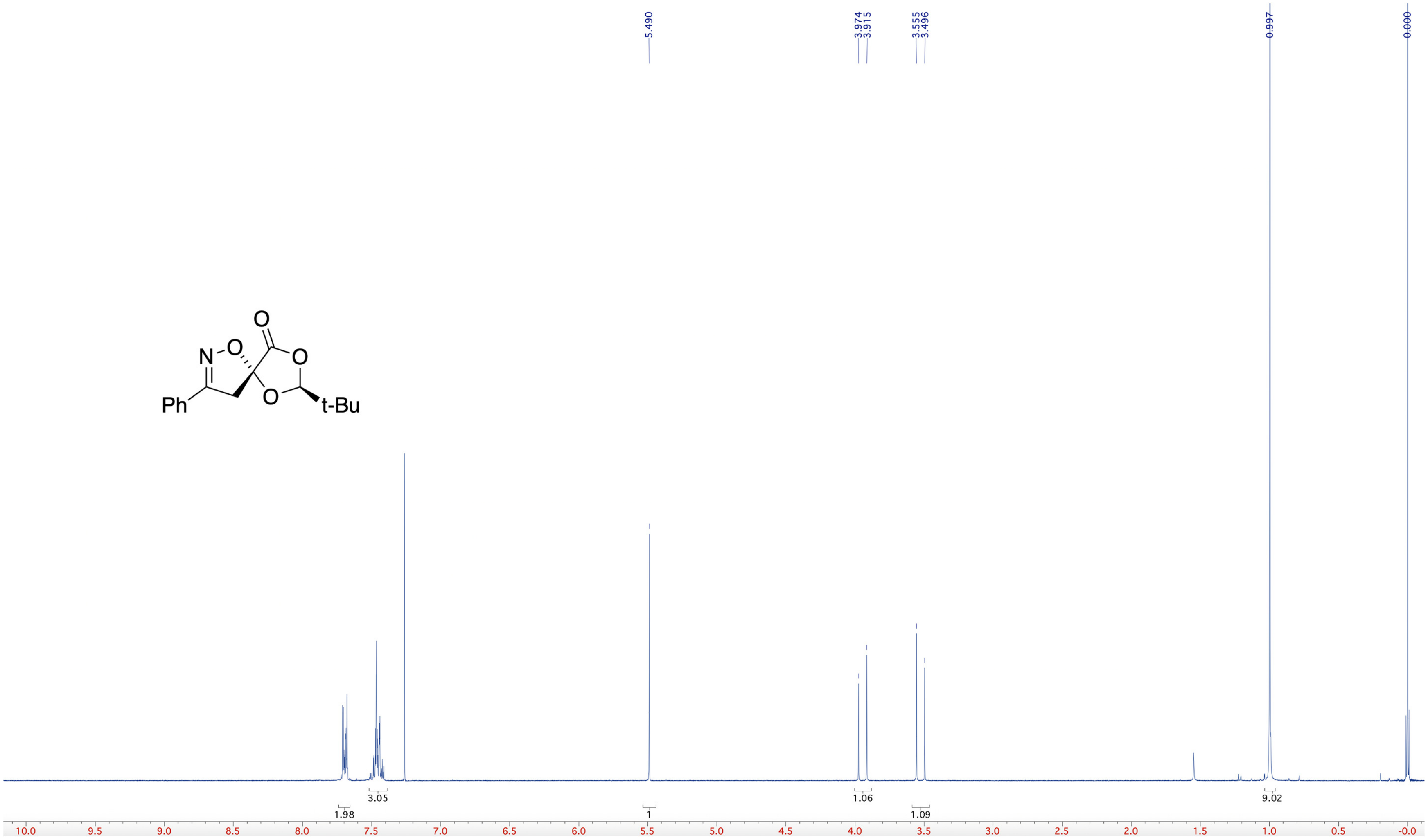

Figure S2. 75 MHz DEPTQ ^{13}C NMR spectrum of **7**

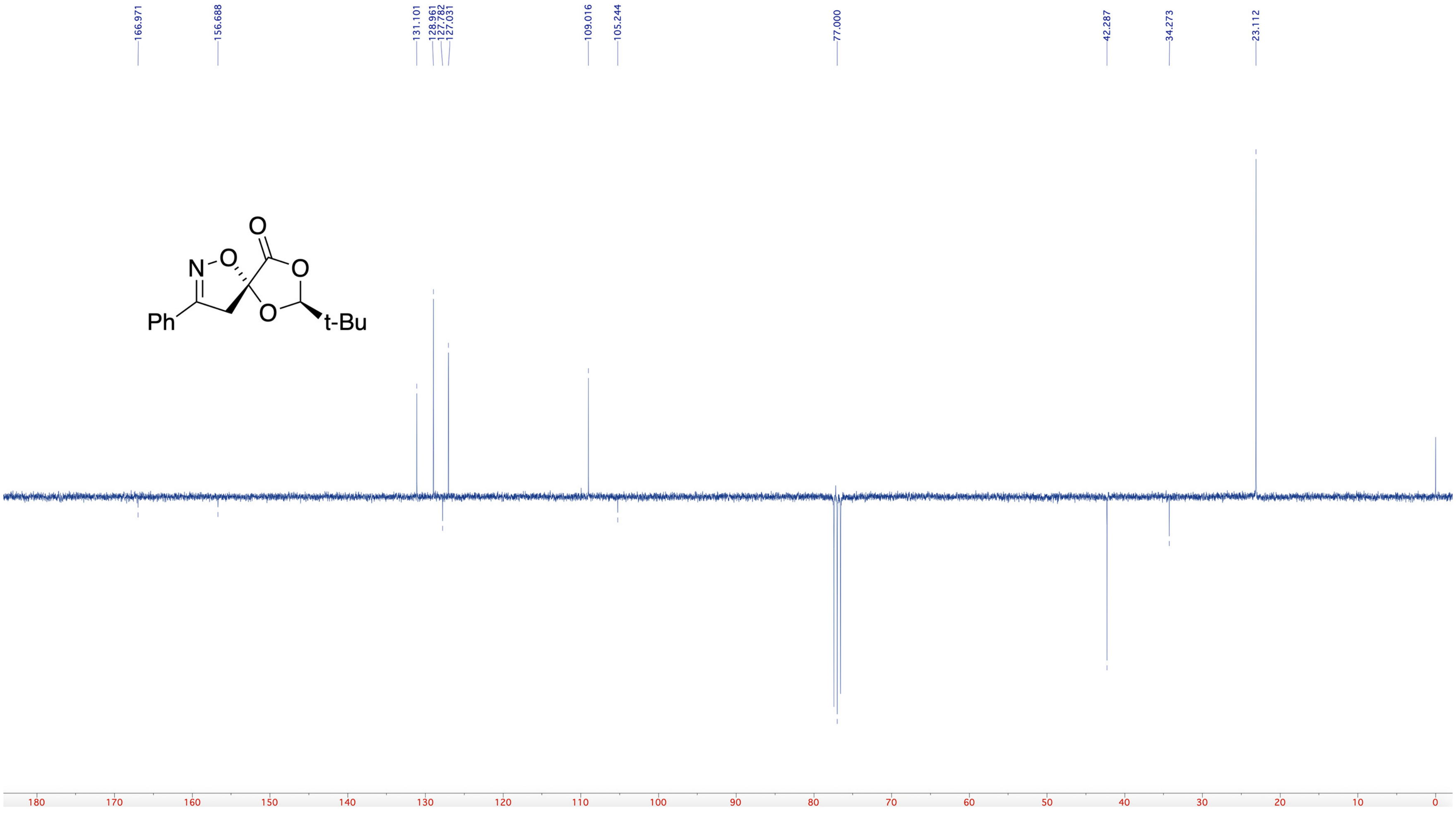

Figure S3. 300 MHz ^{1}H NMR spectrum of **8**

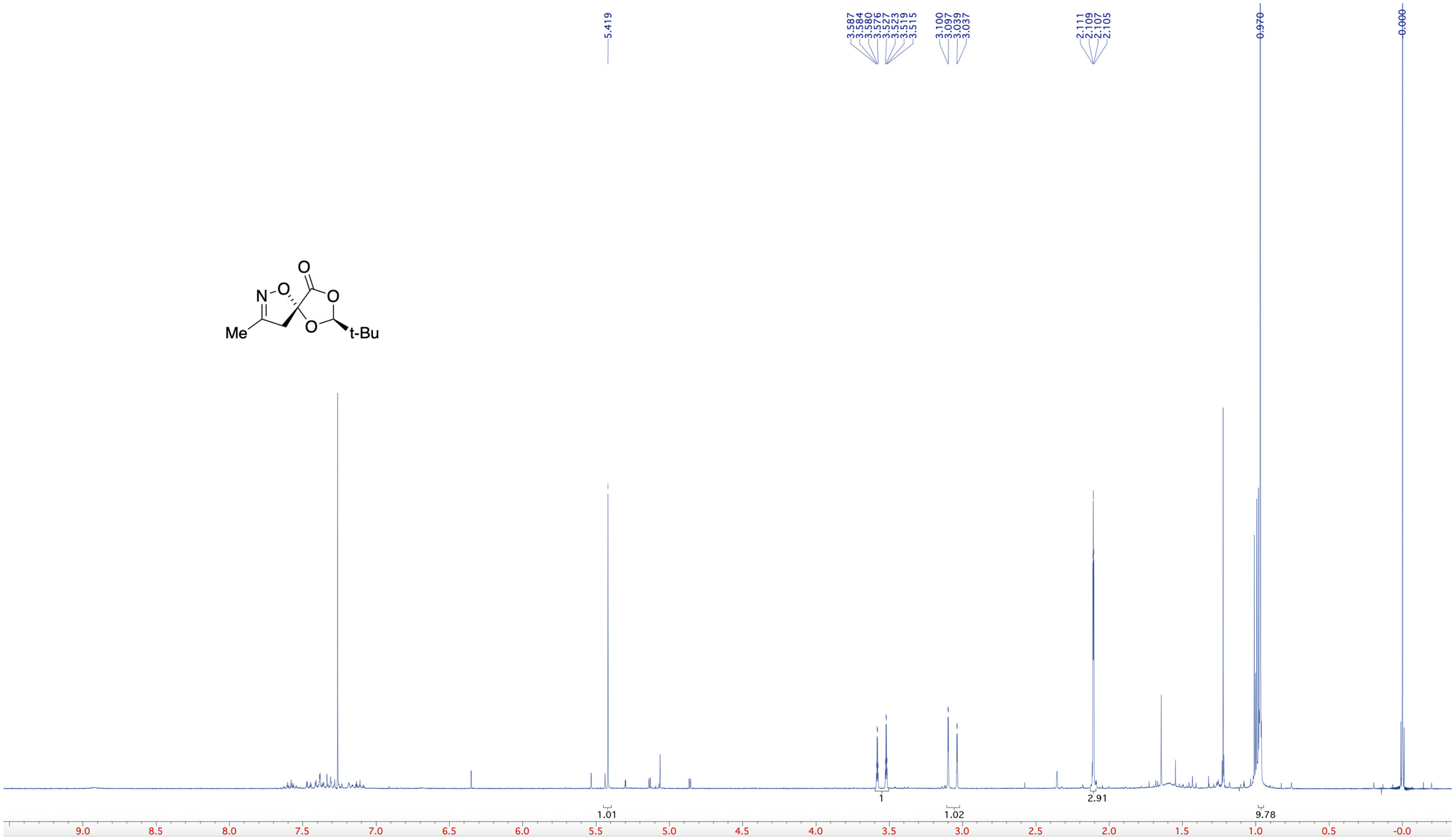

Figure S4. 75 MHz DEPTQ ^{13}C NMR spectrum of **8**

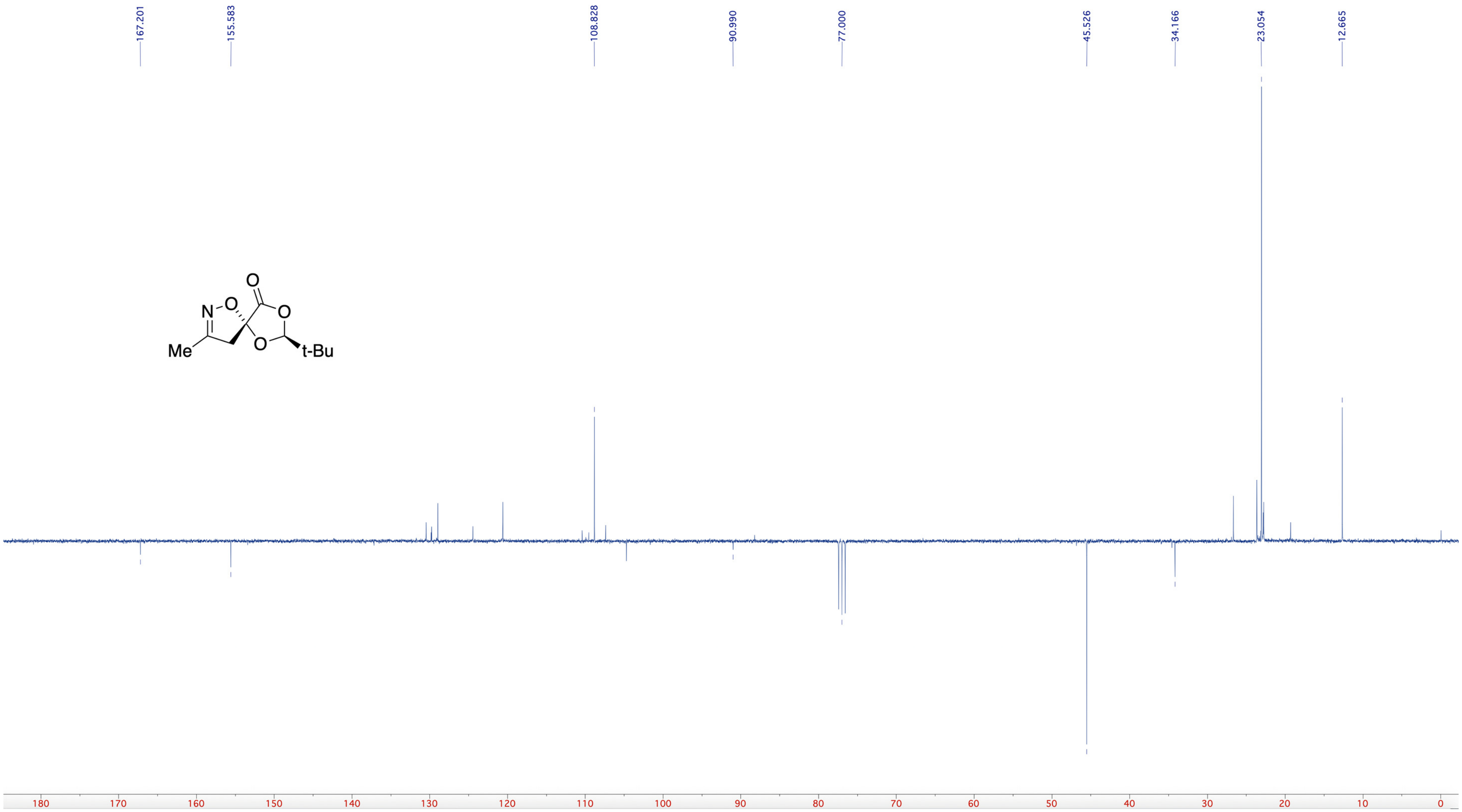

Figure S5. 300 MHz ^{1}H NMR spectrum of **9**

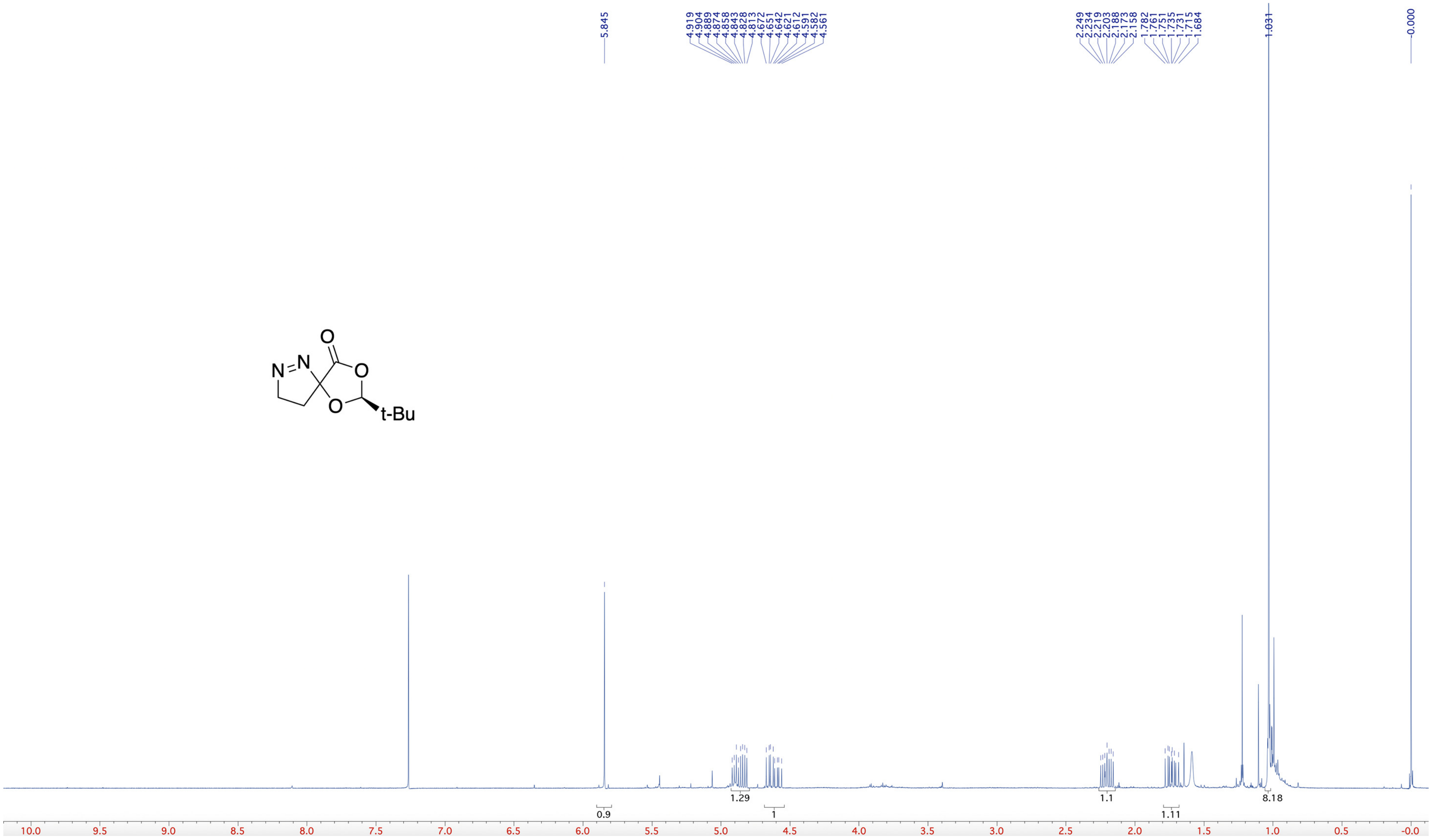

Figure S6. 75 MHz DEPTQ ^{13}C NMR spectrum of **9**

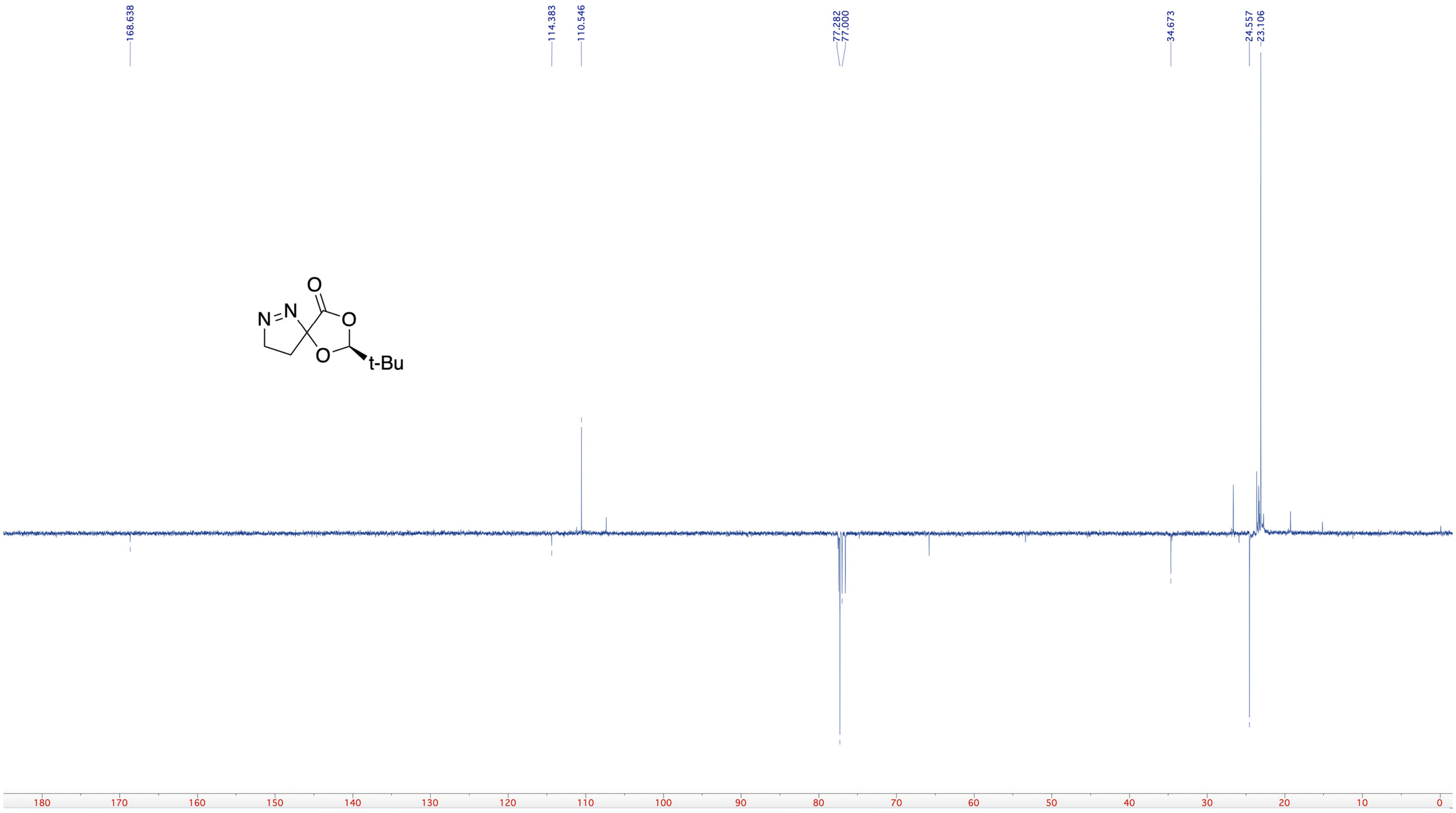

Figure S7. 300 MHz ^{1}H NMR spectrum of **10**

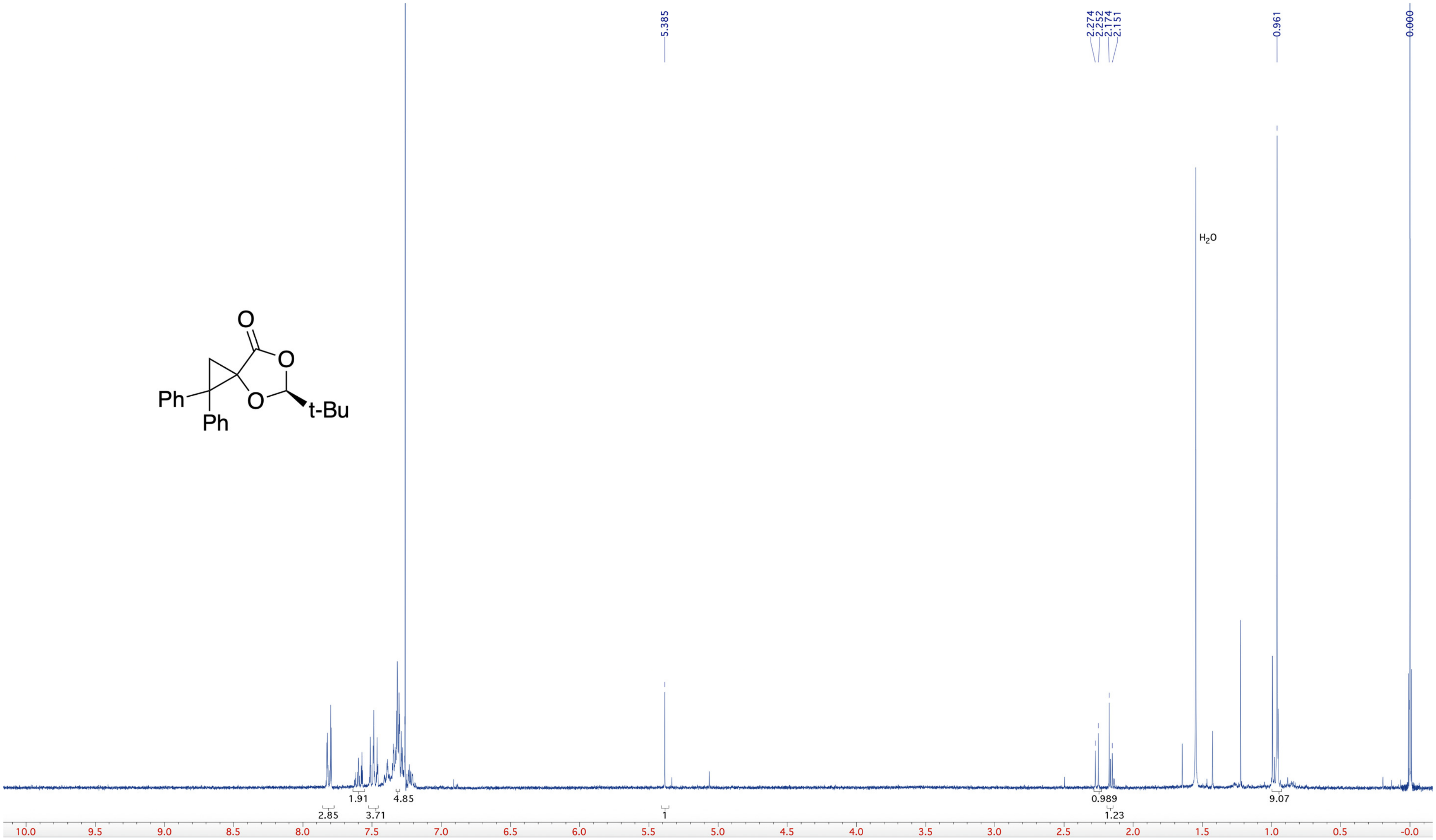

Figure S8. 75 MHz DEPTQ ^{13}C NMR spectrum of **10**

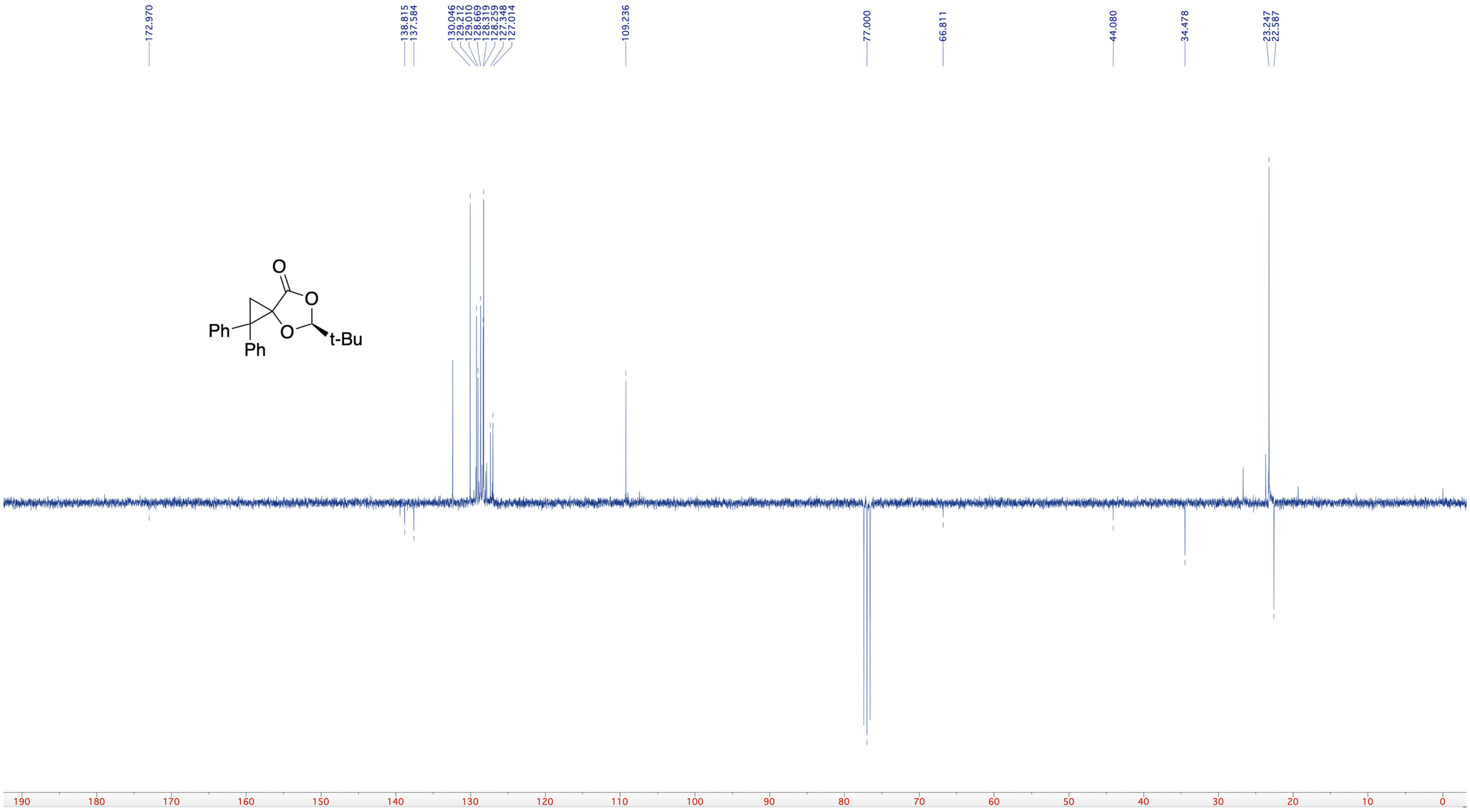

Figure S9. 300 MHz ^{1}H NMR spectrum of pyrolysate from **7**

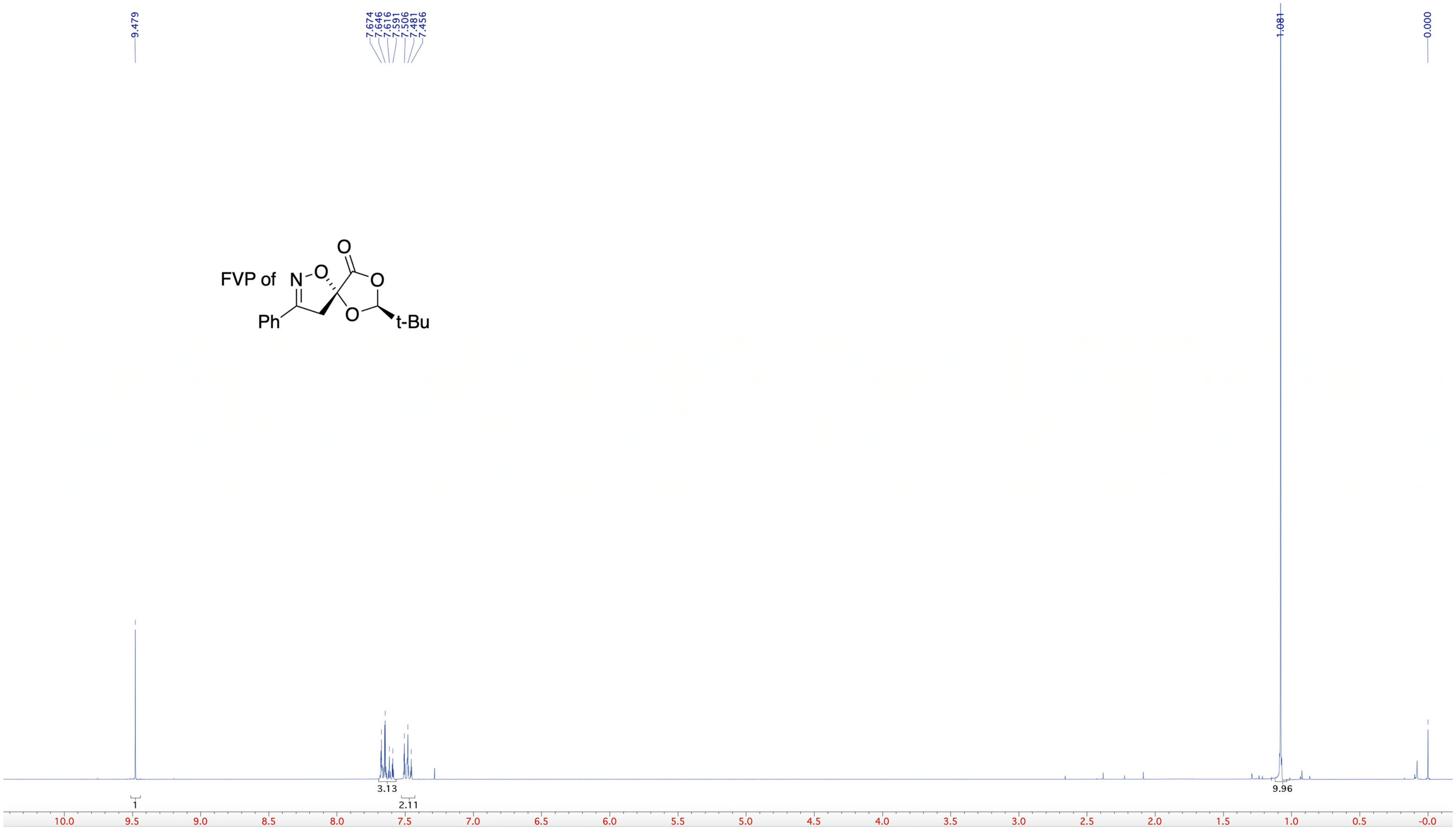

Figure S10. 75 MHz DEPTQ ^{13}C NMR spectrum of pyrolysate from **7**

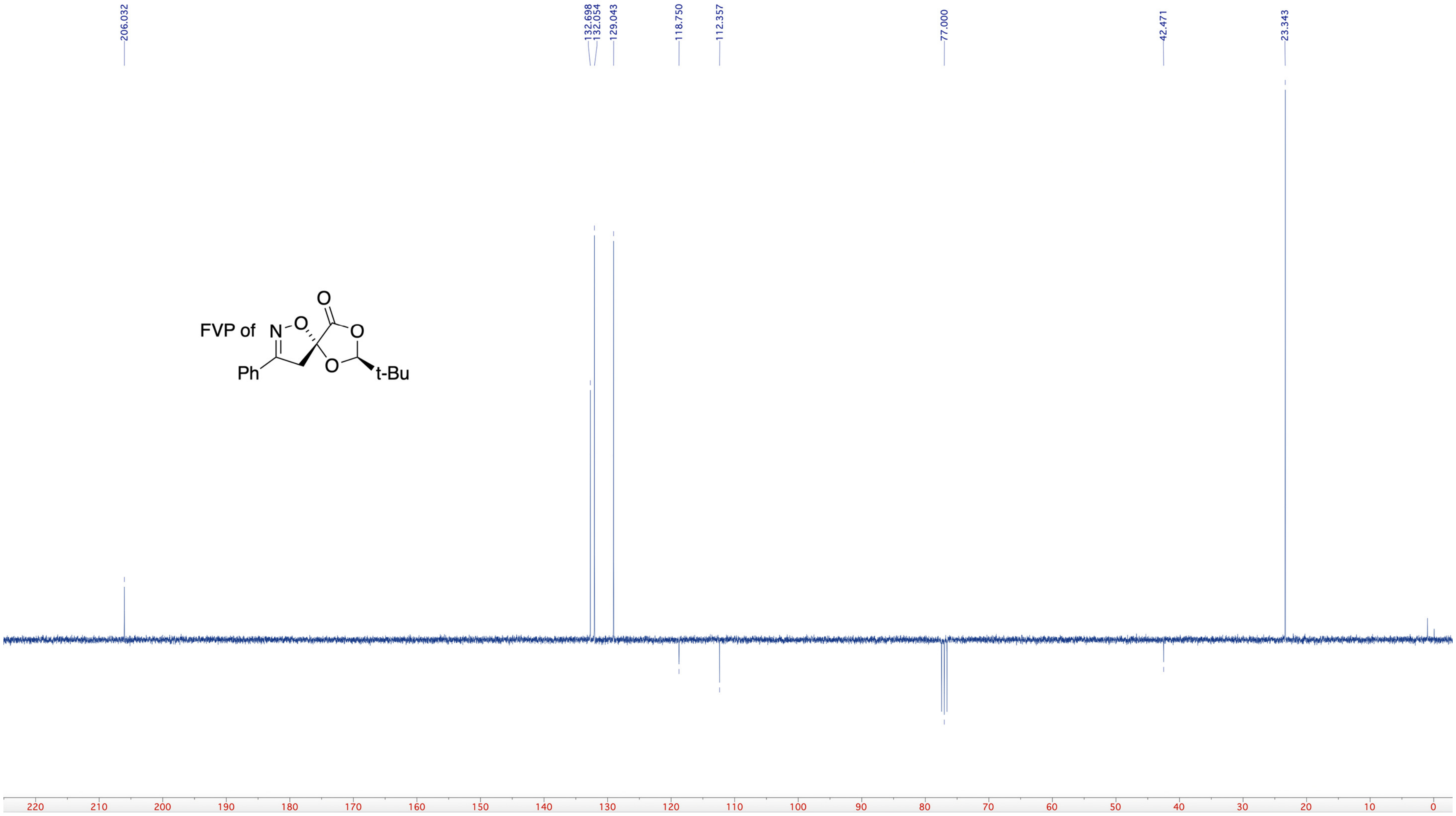

Figure S11. 300 MHz ^{1}H NMR spectrum of pyrolysate from **7** with added EtOH, CH_2Cl_2 and $ClCH_2CH_2Cl$

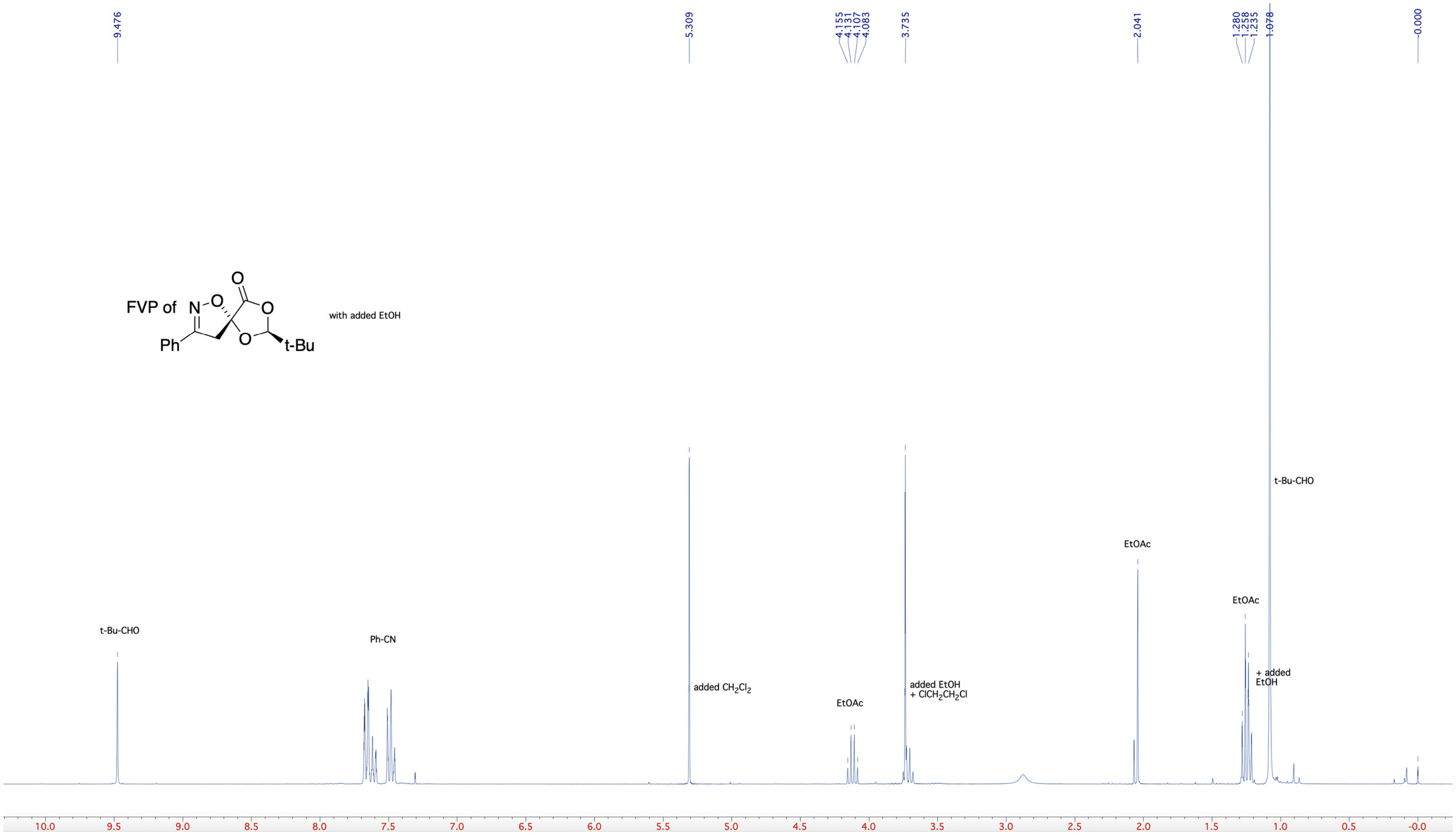

Figure S12. 300 MHz ^1H NMR spectrum of pyrolysate from **8** with added EtOH, CH_2Cl_2 and $ClCH_2CH_2Cl$

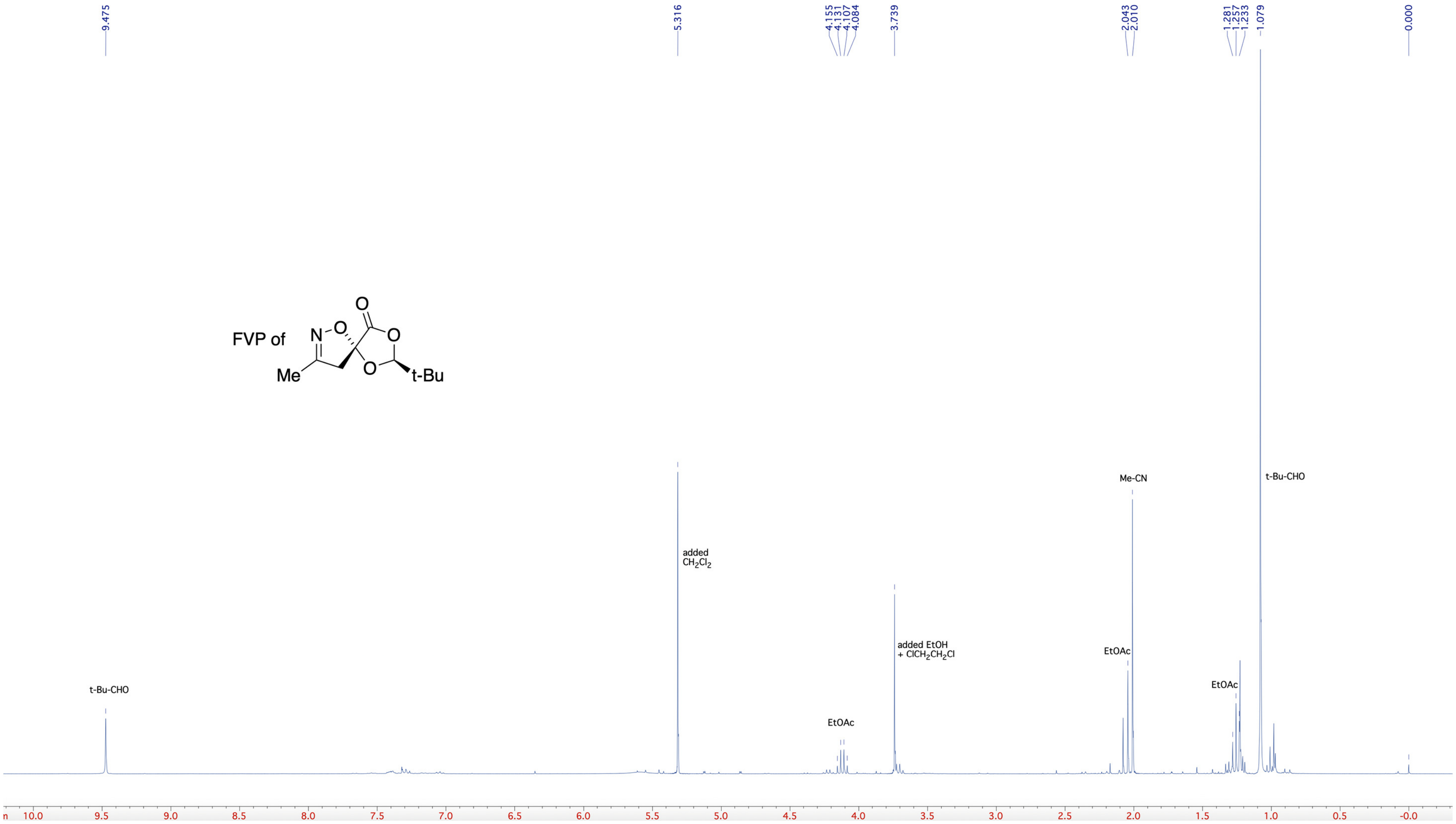

Figure S13. 300 MHz ^{1}H NMR spectrum of pyrolysate from **9** containing **15**

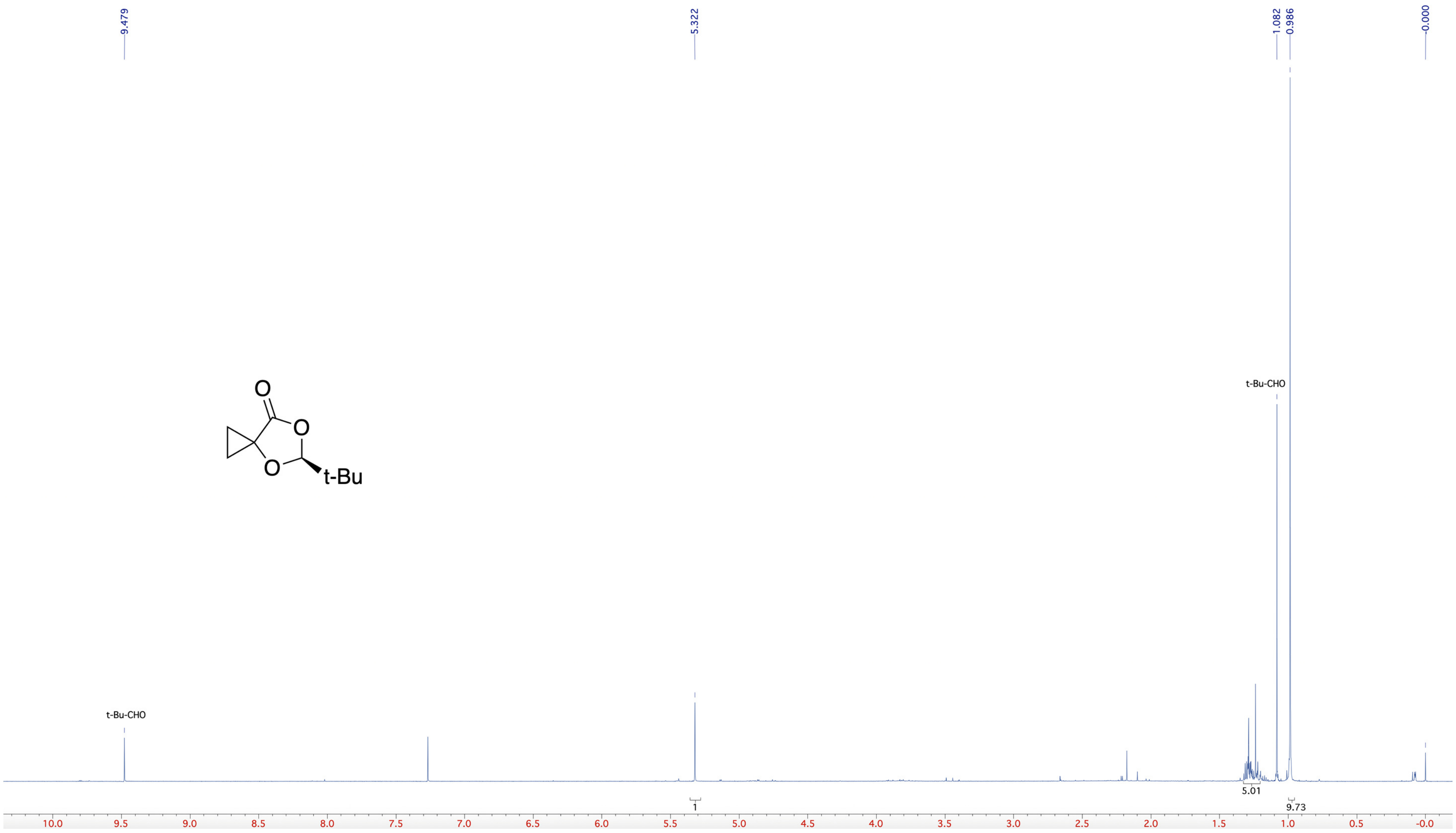

Figure S14. 75 MHz DEPTQ ^{13}C NMR spectrum of pyrolysate from **9** containing **15**

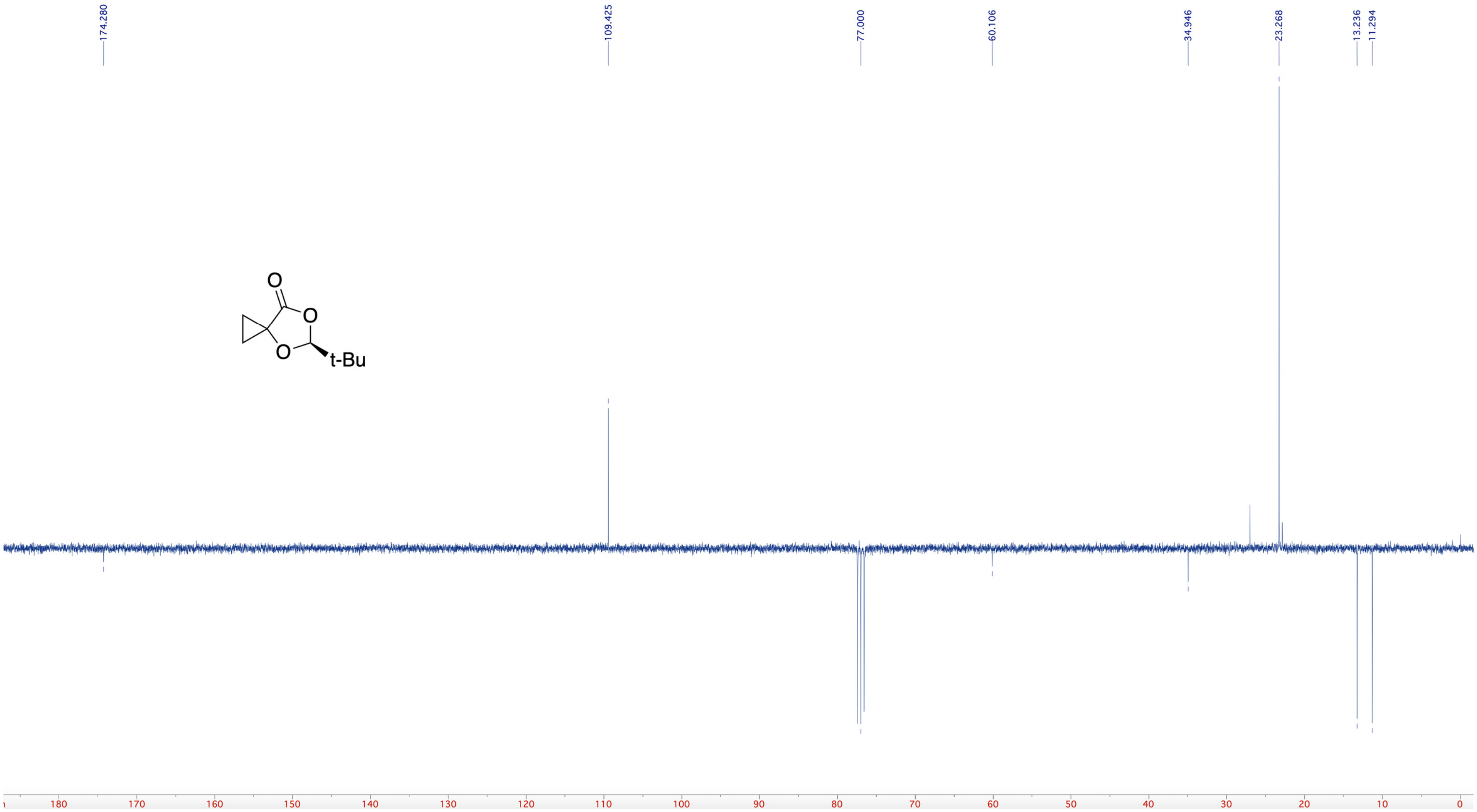

Figure S15. 300 MHz ^{1}H NMR spectrum of pyrolysate from **10** (Fraction 1 from preparative TLC showing signals due to **18**)

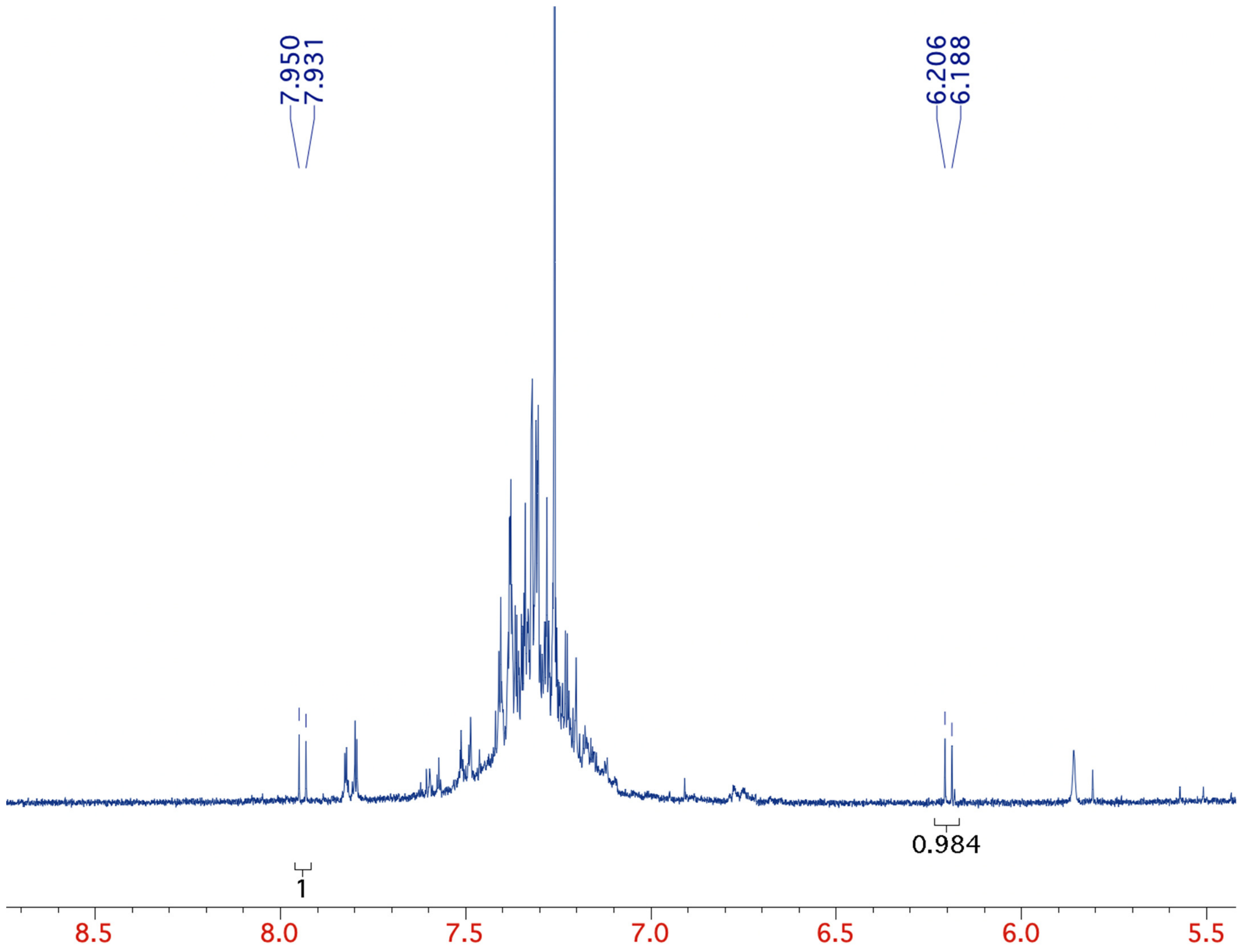

Figure S16. 400 MHz ^{1}H NMR spectrum of pyrolysate from **10** (Fraction 2 from preparative TLC)

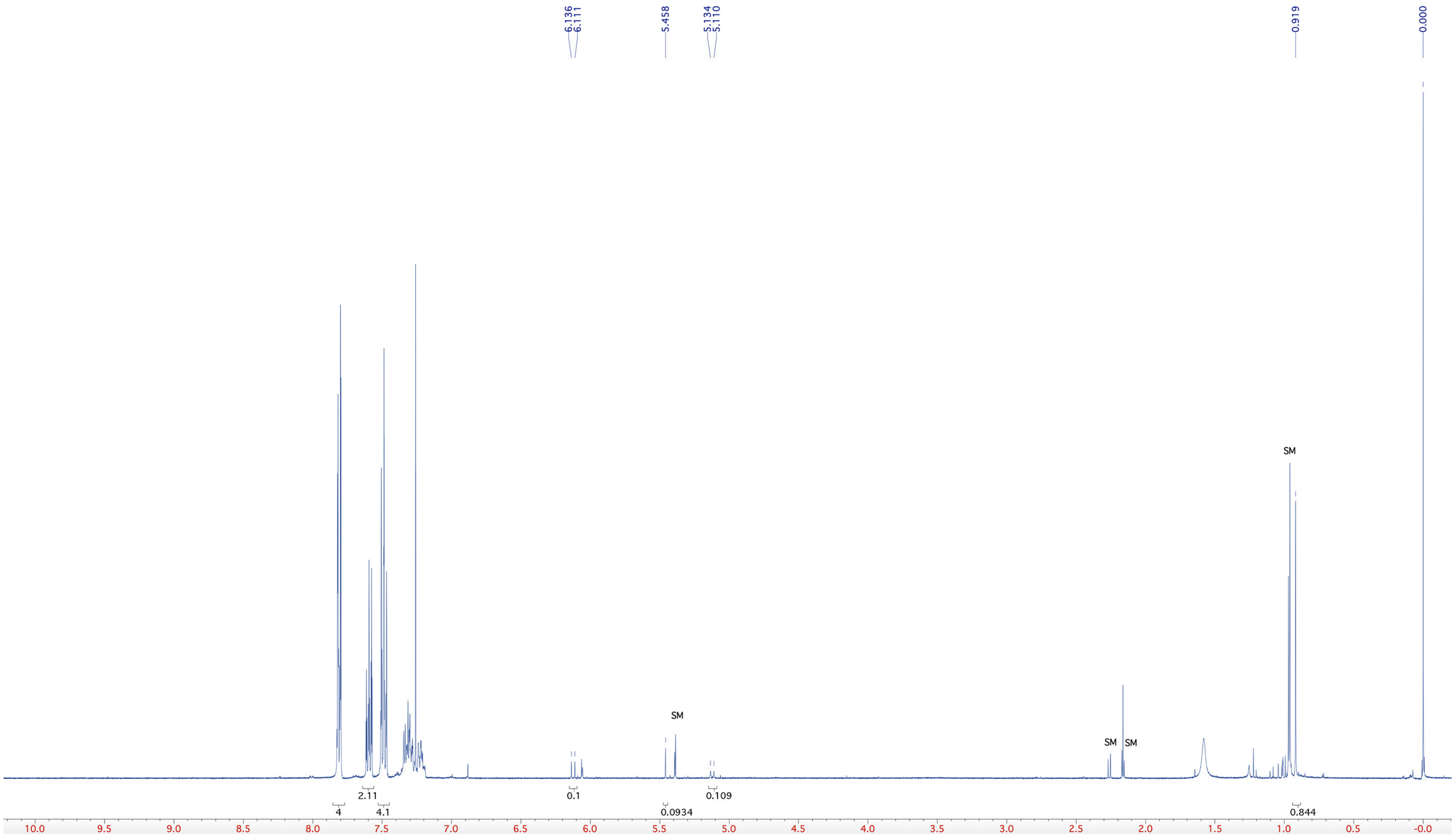

Figure S17. 100 MHz DEPTQ ^{13}C NMR spectrum of pyrolysate from **10** (Fraction 2 from preparative TLC)

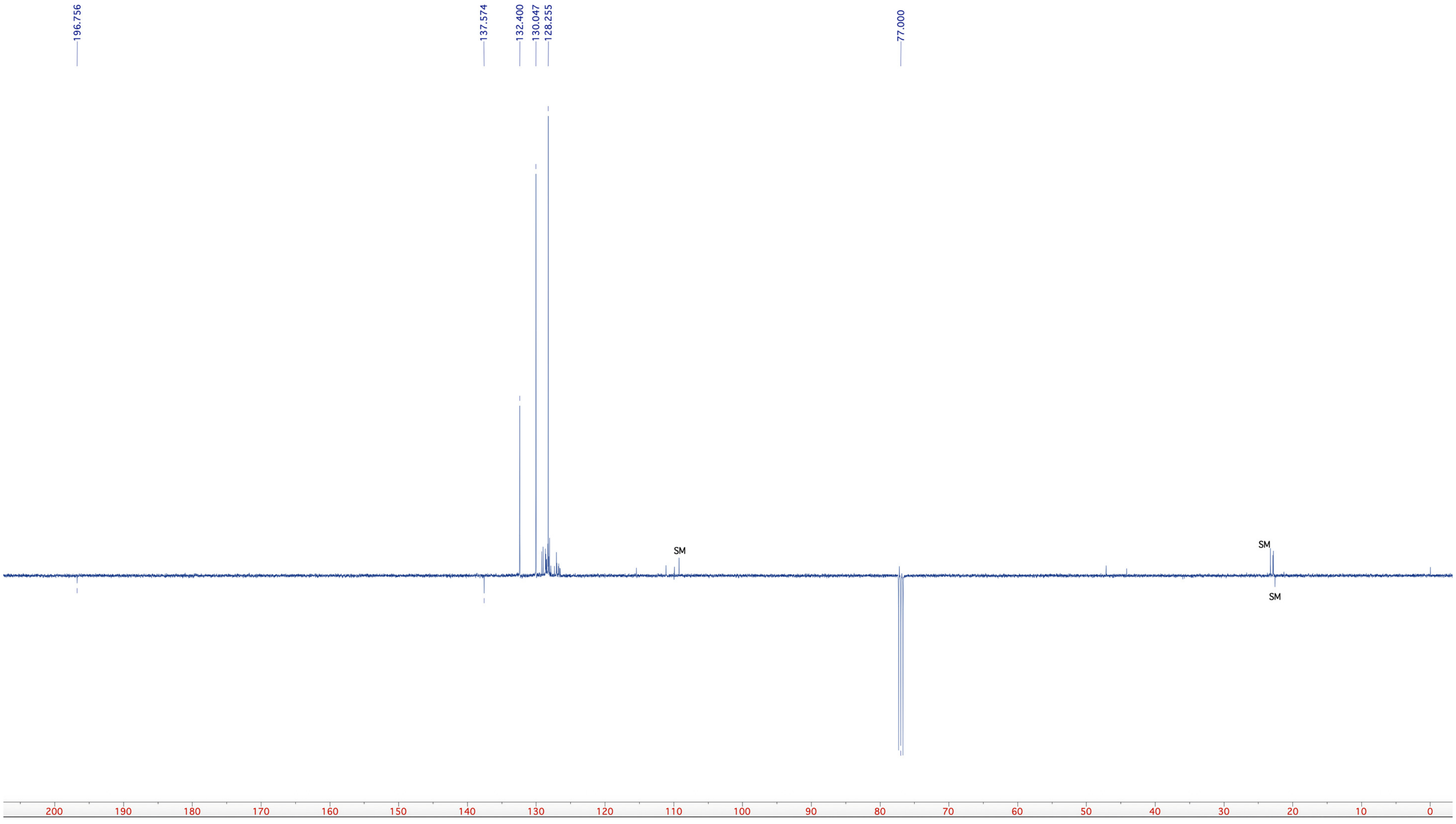

Supplement: Supplementary file 1 [file molecules-30-01246-s001.zip › LAP suppl rev/25Molecules_LAP_suppl.pdf]
